# Contributions of *HFE* polymorphisms to brain and blood iron load, and their links to cognitive and motor function in healthy adults

**DOI:** 10.1002/npr2.12197

**Published:** 2021-07-21

**Authors:** Grégoria Kalpouzos, Francesca Mangialasche, Farshad Falahati, Erika J Laukka, Goran Papenberg

**Affiliations:** ^1^ Department of Neurobiology, Care Sciences and Society Aging Research Center Karolinska Institutet and Stockholm University Stockholm Sweden; ^2^ Division of Clinical Geriatrics Department of Neurobiology, Care Sciences and Society Center for Alzheimer Research Karolinska Institutet Stockholm Sweden; ^3^ Stockholm Gerontology Research Center Stockholm Sweden

**Keywords:** aging, blood, brain, C282Y, cognition, H63D, *HFE* gene, iron, QSM

## Abstract

**Background:**

Brain iron overload is linked to brain deterioration, and cognitive and motor impairment in neurodegenerative disorders and normal aging. Mutations in the *HFE* gene are associated with iron dyshomeostasis and are risk factors for peripheral iron overload. However, links to brain iron load and cognition are less consistent and data are scarce.

**Aims and methods:**

Using quantitative susceptibility mapping with magnetic resonance imaging, we investigated whether C282Y and H63D contributed to aging‐related increases in brain iron load and lower cognitive and motor performance in 208 healthy individuals aged 20‐79 years. We also assessed the modulatory effects of *HFE* mutations on associations between performance and brain iron load, as well as peripheral iron metabolism.

**Results:**

Independent of age, carriers of either C282Y and/or H63D (*HFE*‐pos group, n = 66) showed a higher load of iron in putamen than non‐carriers (*HFE*‐neg group, n = 142), as well as higher transferrin saturation and lower transferrin and transferrin receptors in blood. In the *HFE*‐neg group, higher putaminal iron was associated with lower working memory. In the *HFE*‐pos group, higher putaminal iron was instead linked to higher executive function, and lower plasma transferrin was related to higher episodic memory. Iron‐performance associations were modest albeit reliable.

**Conclusion:**

Our findings suggest that *HFE* status is characterized by higher regional brain iron load across adulthood, and support the presence of a modulatory effect of *HFE* status on the relationships between iron load and cognition. Future studies in healthy individuals are needed to confirm the reported patterns.

## Abstract 2

This study investigated the contribution of genetic polymorphisms in the HFE gene (C282Y and H63D) on blood and brain iron load, and their relationships with cognition, in a healthy sample of adults. The findings indicated that carriers of C282Y and/or H63D displayed higher iron load in putamen and higher transferrin saturation in blood. Results further suggested that in carriers, higher iron load may be beneficial for cognitive performance, independent of age.

## Graphical abstract

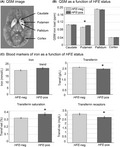

## 1 INTRODUCTION

Iron is an essential metal for numerous biological mechanisms in the brain, where it contributes, for example, to myelin and neurotransmitter syntheses. However, disruption of iron homeostasis can lead to an overload of free iron, which has been linked to cellular dysfunction and destruction via oxidative stress and inflammatory mechanisms.^1^ Neurodegenerative disorders and normal aging are characterized by brain iron overload. Adverse effects of higher brain iron load have been observed in normal aging, such as atrophy, brain dysfunction, and poorer motor and cognitive performance.^2^, ^3^, ^4^, ^5^, ^6^, ^7^, ^8^, ^9^

Iron‐regulating genes may contribute to interindividual variations in markers of iron metabolism. Among the identified iron genes, *HFE* is one of the most studied. *HFE* encodes the HFE protein (high Fe2+, or human homeostatic iron regulator protein), which is involved in hepcidin regulation and iron storage mechanisms.^10^ Two single nucleotide polymorphisms (SNPs) located in the *HFE* gene, the C282Y (rs1800562, risk allele: A) and H63D (rs1799945; risk allele: G) mutations, account for most cases of hereditary hemochromatosis. Individuals with the C282Y mutation, and notably homozygotes, are at higher risk for hereditary hemochromatosis, and both mutations were related to neurodegenerative disorders, including Alzheimer's disease.^10^, ^11^, ^12^, ^13^ Whereas both mutations have been associated with variations of blood markers of iron metabolism (ie, higher iron, ferritin and transferrin saturation, lower transferrin, and transferrin receptors), C282Y has typically shown stronger associations than H63D.^14^, ^15^, ^16^, ^17^, ^18^ A recent study in a Swedish sample of blood donors showed that H63D heterozygotes did not display higher transferrin saturation or ferritin than non‐carriers, but both C282Y homo‐ and heterozygotes were more likely to display elevated values compared to non‐carriers.^19^

The influence of iron‐related genetic polymorphisms has been little studied in relation to brain iron load in gray matter. The few existing studies where C282Y and/or H63D *HFE* variants were tested yielded mixed results.^20^, ^21^, ^22^, ^23^, ^24^ This could be due to different methods of iron quantification and considerably varying sample sizes and sample composition across studies. To our knowledge, only one study examined potential associations between brain iron and cognitive performance as a function of iron‐gene profile: Only non‐carriers of either H63D or TfC2 (transferrin gene, rs1049296) showed a negative association between iron load in basal ganglia and working‐memory performance.^25^ This seems counterintuitive, as carriers of alleles associated with already high iron load would be expected to show lower cognitive performance with further increasing iron load.

Motivated by the scarcity of data and mixed findings, we aimed to determine the potential influence of carrying the *HFE* H63D and/or C282Y mutations (*HFE*‐pos thereafter as opposed to *HFE*‐neg, that is, non‐carriers) on brain iron load, blood markers of iron metabolism, and relationships between brain and blood iron and cognitive/motor performance in healthy volunteers aged 20‐79 years who have not received a clinical diagnosis of iron‐related metabolic disorders. We first hypothesized that the *HFE*‐pos group would display elevated brain and blood iron. Second, we tested whether genetic effects on brain and cognition are magnified in older age due to more constraint neural resources, as predicted by the resource‐modulation hypothesis.^26^ More specifically, we expected stronger effects of the *HFE*‐pos group on brain and blood iron in older age. Finally, we explored relationships between brain iron, blood markers of iron, and cognitive/motor performance encompassing five domains (working memory, executive function, episodic memory, perceptual speed, motor speed). Given the detrimental effects of brain iron overload on cognition,^1^, ^6^, ^27^ we expected that high iron load would be more strongly associated with worse performance in the *HFE*‐pos than in the *HFE*‐neg group. However, given the counterintuitive results reported above,^25^ we could not rule out an alternative hypothesis, with a negative association between iron and cognition in the *HFE*‐neg group, but not in *HFE*‐pos group.

## 2 METHODS

The IronAge study was approved by the Regional Ethical Review Board in Stockholm (number 2016/457‐31/2) and conformed with provisions of the Declaration of Helsinki. The protocol consisted of three visits, with blood sampling, cognitive testing, and magnetic resonance imaging (MRI) assessment.

### 2.1 Participants

Two hundred and thirty‐two individuals were recruited through advertisements in newspapers and student websites. Twenty‐four individuals were excluded due to incomplete data (N = 15 dropped out before MRI) and incidental brain abnormalities (N = 9). The final sample was composed of 208 individuals (Table 1).

**TABLE 1:** Sample characteristics

Variables (Mean ± SE)	*HFE*‐neg (N = 142)	*HFE*‐pos (N = 66)
Age (years)	50.31 ± 1.50	49.06 ± 1.96
Sex (Female/Male)	72/70	36/30
Education (years)	16.36 ± 0.29	15.35 ± 0.38*
MMSE (max 30)	28.43 ± 0.12	28.39 ± 0.15
Body Mass Index	24.7 ± 0.29	24.30 ± 0.36
Smoking % (Never/Former/Current)	59.9/33.8/6.3	56.1/37.9/6.1
Alcohol intake % (No/Moderate/Heavy)	23.9/64.1/12.0	28.8/60.6/10.6
Blood pressure systolic (mmHg)	122.3 ± 1.42	121.2 ± 2.43
Blood pressure diastolic (mmHg)	82.2 ± 0.79	79.4 ± 1.45^tr^
Blood markers		
CRP^a^ (mg/L)	1.23 ± 0.16	1.19 ± 0.17
Iron (mmol/L)	19.44 ± 0.60	21.24 ± 0.88^tr^
Transf (g/L)	2.58 ± 0.03	2.42 ± 0.04**
Transf‐sat (%)	0.31 ± 0.01	0.36 ± 0.02**
Transf‐rec (mg/L)	3.10 ± 0.11	2.76 ± 0.11
Ferr (µg/L)	117.17 ± 8.27	124.76 ± 13.30
Brain iron and volume		
QSM caudate (ppm)	0.108 ± 0.002	0.108 ± 0.002
QSM putamen (ppm)	0.095 ± 0.002	0.104 ± 0.003*
QSM pallidum (ppm)	0.190 ± 0.003	0.187 ± 0.004
QSM cortex^b^ (ppm)	0.041 ± 0.001	0.042 ± 0.001
Volume^c^ caudate (mm^3^)	6887 ± 61.86	7005 ± 98.52
Volume putamen (mm^3^)	8905 ± 75.21	8987 ± 106.72
Volume pallidum (mm^3^)	3646 ± 28.98	3656 ± 40.27
Volume cortex^b^ (mm^3^)	481 333 ± 2360.51	478 683 ± 3644.39
Cognitive and motor functions		
T‐Working memory^d^	50.02 ± 0.74	50.75 ± 1.09
T‐Executive function	50.04 ± 0.81	50.56 ± 1.19
T‐Episodic memory	49.73 ± 0.70	51.48 ± 1.03
T‐Perceptual speed	50.29 ± 0.73	50.26 ± 1.08
T‐Motor speed	49.88 ± 0.67	51.11 ± 0.99

Note

Estimated marginal means from the ANCOVAs are shown for the blood markers of iron metabolism, brain iron, and motor and cognitive performance. All other comparisons were performed using independent‐sample t tests (age, education, MMSE, CRP, and brain volumes) and chi‐square test (sex distribution). Statistical significance: *P < .05; **P < .01; tr: trend .05 < *P* < .10.

Abbreviations: MMSE, Mini‐Mental State Examination; ppm, parts per million; SE, standard error.

^a^ C‐reactive protein.

^b^ cortex excluding the medial temporal lobe.

^c^ volumes are adjusted for total intracranial volume.

^d^ unit‐weighted composite scores transformed into T‐scores metric.

None of the participants reported any current or past neurological or psychiatric conditions (individuals who were diagnosed with depression and/or were taking antidepressants were excluded if it encompassed a period of 2 years prior to inclusion in the study), and none was taking any psychoactive medication or had substance abuse. Other exclusion criteria concerned ineligibility for MRI (presence of metal in the body, claustrophobia), diagnosed conditions with iron deficiency (eg, anemia) or overload (eg, hemochromatosis), cancer, diabetes, dementia, presence of cognitive/memory complaints, surgery (head, heart, eyes, ears), ulcer, Crohn's disease, HIV, hepatitis, and restless leg syndrome.

The sample was divided into three age groups for the purpose of the analyses, based on our previous study where we identified optimal cutoffs according to the non‐linear distribution of iron load in striatum over the adulthood in the same sample.^9^ The younger group was aged 20‐39 (N = 66), the middle‐aged group was aged 40‐59 (N = 69), and the older group was aged 60‐79 (N = 73).

### 2.2 Blood sampling

Venous blood was collected before 10 AM while fasting since 8 PM the day before. Serum, plasma, and Li‐Heparin samples were brought to the Centre for Clinical Laboratory Studies for immediate analyses (Karolinska Hospital, Stockholm), and DNA extraction was performed at the Biobank at Karolinska Institutet.

Using standard procedures, the following blood markers for iron metabolism were measured: plasma iron (Iron), plasma transferrin (Transf), serum transferrin receptors (Transf‐rec), serum ferritin (Ferr), and plasma transferrin saturation (Transf‐sat). In addition, C‐reactive protein (CRP) was assessed as a general marker of inflammation.

### 2.3 Genotyping

DNA samples were transferred on PCR plates and sent to the SNP&SEQ Technology Platform, Uppsala University (National Genomics Infrastructure [NGI], SciLifeLab Sweden). The genotyping was performed using a multiplexed primer extension (SBE) chemistry of the iPLEX assay with detection of the incorporated allele by mass spectrometry with a MassARRAY analyzer from Agena Bioscience.^28^, ^29^, ^30^ Raw data from the mass reader were converted to genotype data using the Typer software (Agena Bioscience). Both *HFE* C282Y (rs1800562) and H63D (rs1799945) genotype distributions were in Hardy‐Weinberg equilibrium (*P*s > .1).

Given the limited number of individual carriers of H63D or C282Y, we pooled the participants in 2 groups: non‐carriers of any of the mutations (*HFE*‐neg) and carriers of either the H63D and/or C282Y (*HFE*‐pos).

### 2.4 MRI acquisition and preprocessing

#### 2.4.1 Acquisition

Participants were scanned on a GE Discovery MR750 3.0T scanner, equipped with an 8‐channel phased array receiving coil, at the MR Center of Karolinska Hospital in Stockholm. A structural T1‐weighted 3D IR‐SPGR image was obtained with the following parameters: repetition time (TR) = 6.96 ms, echo time (TE) = 2.62 ms, 176 axial slices with slice thickness of 1 mm, in‐plane resolution = 0.94 × 0.94 mm^2^, field of view (FOV) = 24 cm, flip angle = 12°. For brain iron quantification, a 3D multi‐echo gradient recalled echo (meGRE) sequence was used with the following parameters: TR = 27.2 ms, 124 axial slices of 1.2 mm thickness, in‐plane resolution = 0.94 × 0.94 mm^2^, FOV = 24 cm, flip angle = 17°. The first TE was 1.9 ms, and it was followed by 7 consecutive TEs with a constant interval of 3.18 ms between them.

#### 2.4.2 Quantitative susceptibility mapping

**FIGURE 1:**
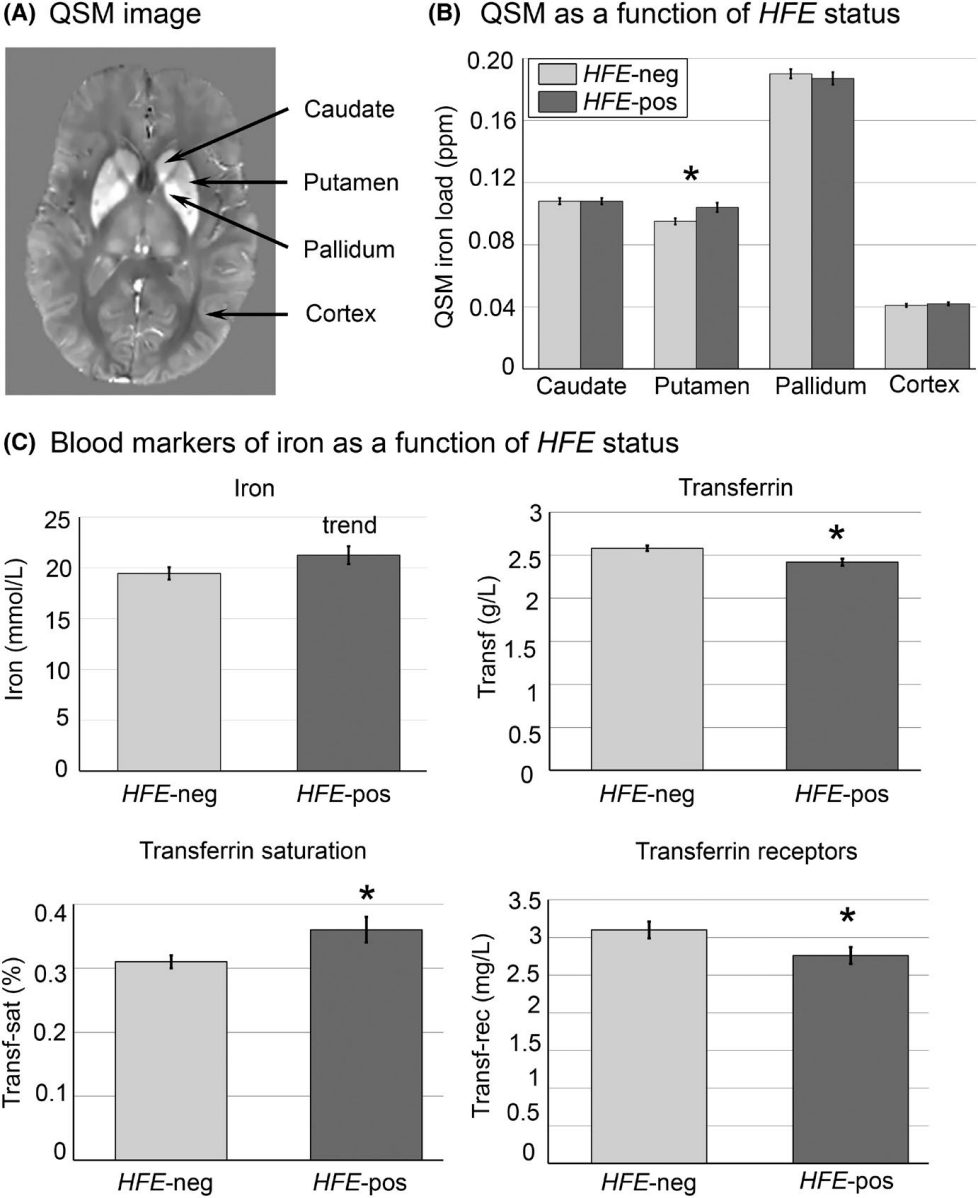
(A) Example of a QSM (quantitative susceptibility mapping) image. Higher signal intensity denotes higher iron load. (B) Iron content in basal ganglia and cortex as a function of *HFE* status (estimated means and standard errors from ANCOVAs). (C) Levels of peripheral Iron, Transferrin, Transferrin saturation and Transferrin receptors as a function of *HFE* status (estimated means and standard errors from ANCOVAs). * indicates significant ANCOVA, *P* < .05. Abbreviation: ppm, parts per million

Initially, the total field map was estimated from the complex meGRE images by performing a non‐linear least square fitting on a voxel‐by‐voxel basis.^31^ The resulting frequency map was then spatially unwrapped using a magnitude image‐guided region growth unwrapping algorithm.^32^ The background fields (the superimposed field contributions that are not caused by the sources inside the brain and mainly generated by air‐tissue interferences) were eliminated using a nonparametric technique based on projection onto dipole fields.^33^ Finally, the corrected frequency map was used as input for the field‐to‐source inverse problem to calculate the map of susceptibility. We used the recommended non‐linear variant of morphology‐enabled dipole inversion (MEDI) method to calculate susceptibility maps.^31^, ^34^ The MEDI Toolbox (http://weill.cornell.edu/mri/pages/qsm.html) was used to generate QSM images (Figure 1A).

Due to the singularity of the dipole kernel at the center of k‐space, the generated QSM images contain relative susceptibility values. Therefore, the QSM images may not necessarily be comparable across subjects in a cohort. A typical approach to address this issue is zero referencing where a common tissue is chosen as a reference and its average susceptibility is subtracted from the susceptibility values of other tissues. We selected a region in left posterior white matter located in the corticospinal tract (MNI coordinates [−24; −27; 39]) as the reference region.^35^ To perform zero referencing on each participant, first the selected coordinate in MNI space was mapped to individual space using a non‐linear registration warp. Using an in‐house developed region‐growing algorithm, a region of 1000 voxels centered on the mapped coordinate was created. The algorithm used the white matter mask to ensure the created region encompassed white matter tissue only. The FMRIB Software Library (FSL, http://fsl.fmrib.ox.ac.uk) was used to calculate non‐linear transformation parameters and to obtain the white matter mask.^36^, ^37^

Automated segmentation of cortical and deep gray matter structures was performed with the Freesurfer image analysis suite—version 6 (http://surfer.nmr.mgh.harvard.edu/) using T1‐weighted images.^38^, ^39^, ^40^ Both QSM images and the segmentation results were resampled to the native structural space, and then, average susceptibility values were regionally calculated on the QSM images, the metric being parts per million (ppm), with higher values indicating higher iron content. Prior to computing statistics, the boundary of segmentations was eroded by one voxel. To avoid the influence of high signal from neighboring vessels and obtain more robust estimates, a fraction (15%) of the most extreme values was removed.^35^

For the analyses, we focused on the basal ganglia (caudate, putamen, pallidum) because iron accumulates most in these regions, and they are relevant for cognitive performance.^2^, ^6^, ^27^ In addition, we added an average measure of cortical iron, which includes all cortical regions of the cerebrum as segmented by Freesurfer, from which we excluded the medial temporal lobe, where artifacts were detected notably in the parahippocampal gyrus. Skewness and kurtosis of the 4 regions of interest were within the acceptable range [−2; 2].

### 2.5 Motor and cognitive performance

A battery of cognitive and psychomotor tests was administered by trained staff, following a standardized procedure. Unit‐weighted composite scores (mean of *z*‐scores) were computed based on the accuracy in these tasks, and these scores were transformed into T‐scores metric. If necessary single scores were inverted so that higher values indicate better performance. Before the composite scores were created, performance for each test was adjusted for test leader and, if appropriate, test version (eg, word list test, binding) and number of attempts (Trail‐Making Test).

#### 2.5.1 Working memory

The composite score for working memory was based on the number correct for 2‐ and 3‐back, as well as number of correct items in the binding task.

##### Numerical n‐back task

A sequence of single numbers appeared on the screen. During every item presentation, subjects indicated whether the digit on the screen was the same as the one shown 1, 2, or 3 digits back. Each digit was shown for 1.5 seconds, with an interstimulus interval of 0.5s. Three blocks for each condition (1‐, 2‐, 3‐back) were performed in sequential order (1‐2‐3; 1‐2‐3). The 1‐back block had 13 items, the 2‐back block had 14 items, and the 3‐back block had 15 items.

##### Binding task

The binding task assessed the ability to associate visuospatial features in working memory.^41^ Five colored uppercase letters were presented in the center of a 5 × 4 grid, accompanied with 5 colored crosses displayed randomly in the other squares of the grid. Participants were asked to remember the associations between the 5 colored letters with the location of the cross of the same color. Each trial started with a fixation cross for 2 seconds, and then, consonants were shown.

Five seconds were allocated to this encoding phase, followed by a retention interval of 8 seconds (fixation cross). Participants had to determine whether a black lowercase letter was presented in the correct location by pressing yes or no. In total, 20 trials were administered.

#### 2.5.2 Episodic memory

The composite score for episodic memory was based on the word list test, as well as cued recall and recognition from the Face‐Name Paired‐Associates Task.

##### Word list test

Word list test comprised a list of 16 unrelated concrete nouns, which were presented both orally and visually with a new word appearing every 5s. Immediately after presentation, participants were given two minutes for oral free recall.^42^

##### Face‐name paired‐associates task

Subjects were shown faces with a fictional first name printed on the right side of the face, forming a face‐name pair. Subjects were instructed to remember the name associated with each face and answer orally when asked. During retrieval, each face was presented together with three letters, of which one corresponded to the first letter of the name that was presented together with the face (32 trials).^43^ Encoding and retrieval stimuli were presented for 4 seconds. There were eight blocks (4 trials) of encoding and retrieval each, and 7 blocks of an interference task where participants were asked to count backward for 15 seconds to prevent rehearsal and minimize the influence of short‐term memory. This was followed by a recognition task (32 trials), where previously presented faces were presented for 7 seconds together with the correct name and two names, of which one was presented in the same block but with a different face and one was new. Subjects had to indicate by button press the correct name. They could also indicate that they did not remember the correct answer. To account for response bias, hits minus false alarms were computed for the recognition task.

#### 2.5.3 Executive functioning

Executive functioning was assessed with a composite score based on the difference in completion time between trail‐making test (TMT)‐B and TMT‐A, as well as the fluency and random generation scores.

##### Trail‐making test

The TMT‐A and TMT‐B versions each consisted of 25 circles with the same distance between them. For TMT‐A, participants were required to connect encircled digits in numerical order (1, 2, 3, etc). For TMT‐B, circles included both digits and letters, and the task was to connect these in alternating order (1‐A, 2‐B, 3‐C, etc).^44^ The test was interrupted by the test leader in case of a mistake and repeated.

##### Letter and category fluency

For the letter fluency tasks, participants were asked to orally generate as many words as possible within 60 seconds, beginning with the letters F and A, respectively. They were instructed that proper names, numbers, or words with a different suffix were not credited. For category fluency, participants were asked to orally generate as many words as possible within 60 seconds, belonging to the categories animals and professions, respectively. The four fluency measures were combined into one average score.

##### Random generation

Participants had to produce a consonant every second (condition 1) or every two seconds (condition 2), whenever a square was presented on the computer screen.^45^ A series of 50 random consonants had to be generated in each condition (ie, no vowels, no alphabetic order or reverse order, no repetitions of random sequences, no spelling of words). Scores were corrected for errors, and the two conditions were averaged into a final score.

#### 2.5.4 Processing speed

The composite score was based on the time in seconds needed to accomplish the TMT‐A and number of correctly copied symbols during 90 seconds.

##### TMT‐A

Participants were instructed to draw lines to connect circled digits (1‐25) in ascending order as rapidly as possible and without lifting the pen.

##### Digit symbol substitution test

Digit Symbol Substitution Test (DSST) is a general index of perceptual speed.^46^ The DSST consists of nine digit‐symbol pairs followed by a list of digits. Under each digit, the participant is required to fill in the corresponding symbol as rapidly as possible during 90s. Errors were subtracted from the total score.

#### 2.5.5 Motor speed

A composite score was created based on the below described two scores from the Purdue Pegboard Test and the main score from the Grooved Pegboard Test.

##### Purdue Pegboard test

Purdue Pegboard Test (Model 32020, Lafayette Instrument) was used to measure fine motor control (finger and hand dexterity).^47^ Participants were instructed to place as many pegs into the peg‐holes as possible within 30 seconds. Three conditions were tested: right hand only, left hand only, and both hands simultaneously. In addition, a condition where pegs and washers had to be assembled was conducted, but not included in the total score. Subsequently, two separate scores were generated: (1) average number of pegs inserted by right and left hands, and (2) number of pegs inserted by both hands.

##### Grooved Pegboard test

The standard apparatus (Lafayette Instruments) was used to assess visual‐motor coordination, motor speed, and fine motor control.^44^ Participants were instructed to place 25 pegs, one at a time, into key‐shaped holes as quickly as possible. The test had two conditions: positioning pegs from left to right on the board using the right hand, and positioning pegs from right to left using their left hand. The score for the right and the left hand (measured in seconds) was averaged and inversed, such that higher values indicate faster speed.

### 2.6 Statistical analyses

To identify genetic association and their potential interactions with age on brain iron load, a multivariate analysis of covariance (MANCOVA) was conducted with regional QSM (caudate, putamen, pallidum, cortex) as dependent variables and age group (younger vs. middle‐aged vs. older) and *HFE* status (*HFE*‐neg vs. *HFE*‐pos) as between‐subjects factors. Sex, education, and regional volumes were included as covariates. Follow‐up analyses of covariance (ANCOVAs) were conducted to identify the significant dependent variables.

To test the genetic association of *HFE* status on blood markers of iron metabolism, a MANCOVA was conducted with five dependent variables (Iron, Transf, Transf‐rec, Transf‐sat, Ferr). As above, age group and *HFE* status were included as between‐subjects factors and sex and education as covariates. Follow‐up ANCOVAs were conducted to identify the significant dependent variables.

To test the genetic association of *HFE* status on motor and cognitive functions, a MANCOVA was conducted with five dependent variables (working memory, episodic memory, executive function, perceptual speed, and motor speed). As above, age group and *HFE* status were included as between‐subjects factors and sex and education as covariates. Follow‐up ANCOVAs were conducted to identify the significant dependent variables.

To determine whether peripheral and brain iron were related, partial correlations were conducted in the entire sample, adjusting for age, sex, education, and regional volume.

Finally, partial correlation analyses (with same covariates as above) were performed to assess whether brain iron load and blood markers of iron metabolism were related to cognitive performance as a function of *HFE* status.

Regarding the correlational analyses, in addition to Bonferroni correction for multiple tests, we conducted bootstrapping analyses to confirm the stability of the effects. The bootstrapping analyses were based on 5000 samples. Thus, we also report the bias‐corrected (95%) confidence intervals (CIs) of parameter estimates for the correlation coefficients. If 95% confidence intervals for the regression coefficients did not include zero, the effects were considered reliable.

Regarding the distribution of the data across the sample, all QSM and cognitive variables, Iron, Transf, and Transf‐sat, were normally distributed. Transf‐rec and Ferr were skewed and were therefore log‐transformed (log10). Skewness and kurtosis were within the acceptable range [−2; 2] except for Transf‐rec(log) due to 3 outliers. Considering the modest sample size, despite the acceptable skewness and kurtosis of the variables of interest, potential remaining outliers were further tracked using a standard deviation of 3.29 as a cutoff (*P* < .001). This approach revealed a negligible number of outliers on the following variables: Iron (N = 2), Transf‐sat (N = 1), Transf (N = 1), QSM cortex (N = 1), perceptual speed (N = 1), and working memory (N = 3). The analyses were rerun after excluding them, and results are reported in the Section 3.8, control analyses.

The statistical analyses were conducted using SPSS for Windows 27 (SPSS).

## 3 RESULTS

### 3.1 Prevalence of C282Y and H63D

Allele frequency for the *HFE* C282Y mutation was 7.7% (A allele), and there were no homozygotes for this SNP. Allele frequency for the *HFE* H63D mutation was 24.5%, two were homozygous (G allele). One individual was heterozygous for both C282Y and H63D (Table 2).

**TABLE 2:** Genotype distributions for *HFE* H63D (rs1799945; C allele = wildtype) and *HFE* C282Y (rs1800562; G allele = wild type) in the whole sample and according to age group in parentheses (younger/middle‐aged/older)

	rs1800562 G/G	rs1800562 A/G	rs1800562 A/A	Total
rs1799945 C/C	142 (44/47/51)	15 (6/4/5)	0	157
rs1799945 C/G	48 (16/16/16)	1 (0/0/1)	0	49
rs1799945 G/G	2 (0/2/0)	0	0	2
Total	192	16	0	208

### 3.2 Age, *HFE* status, and brain iron

The MANCOVA conducted on brain QSM iron showed a significant main effect of age group (Wilks' lambda = 0.65, *F*[8,386] = 11.78, *P* < .001, partial η^2^ = 0.196), a main effect of *HFE* status (Wilks' lambda = 0.89, *F*[4,193] = 6.18, *P* < .001, partial η^2^ = 0.114), but no significant age group X *HFE* interaction (Wilks' lambda = 0.98, *F*[8,386] = 0.63, *P* = .756, partial η^2^ = 0.013).

Further ANCOVAs showed that older adults significantly displayed higher iron levels in all 4 regions (caudate: *F*[2,196] = 7.63, *P* = .001, partial η^2^ = 0.072; putamen: *F*[2,196] = 30.48, *P* < .001, partial η^2^ = 0.237; pallidum: *F*[2,196] = 6.88, *P* = .001, partial η^2^ = 0.066; cortex: *F*[2,196] = 3.44, *P* = .034, partial η^2^ = 0.034).

In these follow‐up ANCOVAs, *HFE* status had only a significant association with iron in putamen, *F*[1,196] = 5.87, *P* = .016, partial η^2^ = 0.029 (for the other regions, *P*s > .05; see Table 1 and Figure 1B), where HFE‐pos was associated with higher iron load. No interactions between age group and *HFE* status were significant on the variables of interest (*P*s > .05).

### 3.3 Age, *HFE* status, and blood markers of iron metabolism

The MANCOVA conducted on blood iron showed a significant main effect of age group (Wilks' lambda = 0.82, *F*[10,392] = 4.13, *P* < .001, partial η^2^ = 0.095), a main effect of *HFE* status (Wilks' lambda = 0.92, *F*[5,196] = 3.52, *P* = .005, partial η^2^ = 0.082), but no significant age group X *HFE* interaction (Wilks' lambda = 0.94, *F*[10,392] = 1.22, *P* = .276, partial η^2^ = 0.030).

Follow‐up ANCOVAs showed that older age was associated with lower Transf, *F*(2,200) = 5.02, *P* = .007, partial η^2^ = 0.048, and higher Ferr(log), *F*(2,200) = 20.87, *P* <.001, partial η^2^ = 0.173, but not with Iron, *F*(2,200) = 0.37, *P* = .69, partial η^2^ = 0.004, Transf‐sat, *F*(2,200) = 1.43, *P* = .24, partial η^2^ = 0.014, and Transf‐rec(log), *F*(2,200) = 1.72, *P* = .18, partial η^2^ = 0.017.

The ANCOVAs further showed a significant effect of *HFE* status on Transf‐sat, *F*(1,200) = 8.04, *P* = .005, partial η^2^ = 0.039, Transf, *F*(1,200) = 8.65, *P* = .004, partial η^2^ = 0.041, and Transf‐rec(log), *F*(1,200) = 8.70, *P* = .018, partial η^2^ = 0.028, a trend on Iron, *F*(1,200) = 2.86, *P* = .09, partial η^2^ = 0.014, and a non‐significant effect on Ferr(log), *F*(1,200) = 0.51, *P* = .474, partial η^2^ = 0.003 (Table 1, Figure 1C). Compared with HFE‐neg, HFE‐pos displayed lower levels of Transf and Transf‐rec and higher levels of Transf‐sat. No age group X *HFE* interactions were significant (*P*s > 0.05).

### 3.4 Age, *HFE* status, and motor and cognitive functions

The MANCOVA conducted on motor and cognitive functions showed a significant main effect of age group where older age was associated with poorer performance (Wilks' lambda = 0.56, *F*[10,392] = 13.08, *P* <.001, partial η^2^ = 0.250). The main effect of *HFE* status was not significant (Wilks' lambda = 0.98, *F*[5,196] = 0.66, *P* = .653, partial η^2^ = 0.017), neither was the age group X *HFE* interaction (Wilks' lambda = 0.97, *F*[10,392] = 0.51, *P* = .880, partial η^2^ = 0.013).

Further ANCOVAs showed that older adults significantly displayed lower performance in all 5 domains (working memory: *F*[2,200] = 22.68, *P* < .001, partial η^2^ = 0.185; episodic memory: *F*[2,200] = 33.50, *P* < .001, partial η^2^ = 0.251; executive function: *F*[2,200] = 5.267, *P* = .006, partial η^2^ = 0.050; perceptual speed: *F*[2,200] = 26.00, *P* < .001, partial η^2^ = 0.206; motor speed: *F*[2,200] = 48.66, *P* < .001, partial η^2^ = 0.327).

### 3.5 Associations between putaminal iron and blood markers of iron metabolism

**FIGURE 2:**
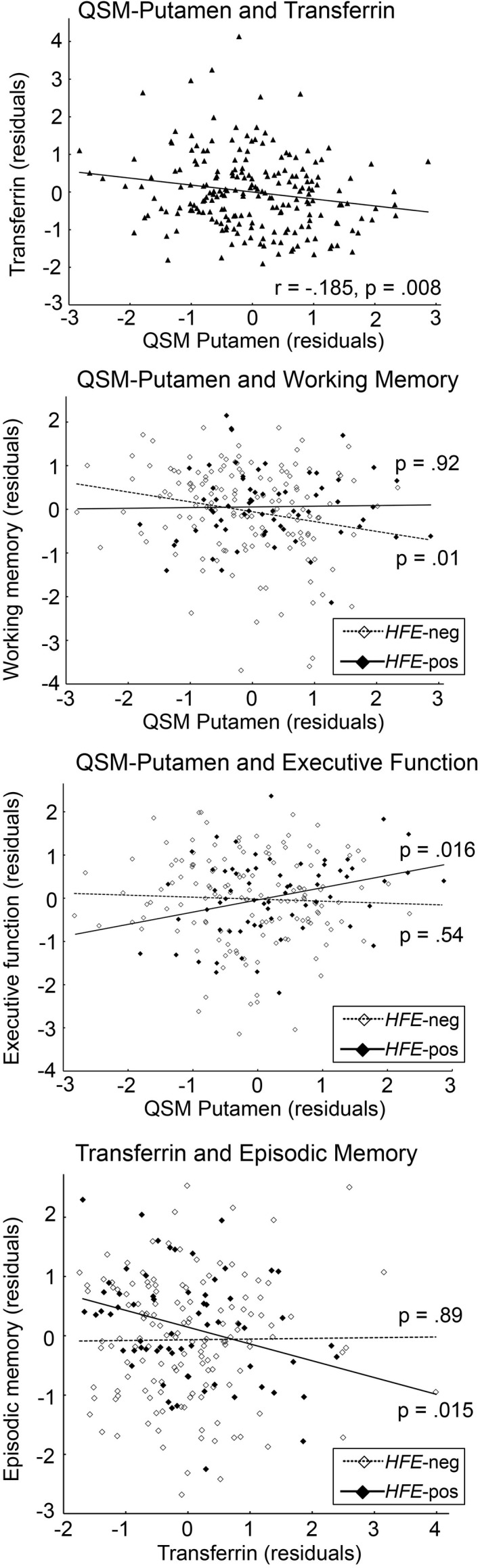
Scatterplots of correlations (from top to bottom) between iron (QSM—quantitative susceptibility mapping) in putamen and Transferrin in the whole sample, iron in putamen and working‐memory performance as a function of *HFE* status, iron in putamen and executive‐function performance as a function of *HFE* status, Transferrin and episodic‐memory performance as a function of *HFE* status. Values were adjusted for age, sex, and education, as well as volume of putamen in the analyses including QSM

QSM putamen was negatively related to Transf in the total sample (*r* = −.185, *P* = .008; bootstrapping 95% CI [−0.301 to −0.062]), positively to Ferr(log) (*r* = .14, *P* = .047; bootstrapping 95% CI [0.017 to 0.262]) and unrelated to Iron (*r* = .009, *P* = .90), Transf‐sat (*r* = .076, *P* = .28), and Transf‐rec(log) (*r* = −.082, *P* = .24) (Table 3, Figure 2). After Bonferroni correction, only the correlation between putaminal iron and Transf could be considered significant (corrected *P* = .05/5 = .01), although the associations involving both Transf and Ferr(log) were estimated reliable based on bootstrapping.

**TABLE 3:** Partial correlations between iron parameters in brain and blood and cognitive and motor function according to *HFE* status

	Working memory	Episodic memory	Executive functioning	Perceptual speed	Motor speed
QSM putamen *HFE*‐neg	*r* = −.21 *P* = .013	*r* = .03 *P* = .745	*r* = −.05 *P* = .540	*r* = −.06 *P* = .514	*r* = .09 *P* = .311
QSM putamen *HFE*‐pos	*r* = .01 *P* = .918	*r* = .21 *P* = .109	*r* = .31 *P* = .016	*r* = .15 *P* = .231	*r* = .04 *P* = .753
Transferrin *HFE*‐neg	*r* = .11 *P* = .218	*r* = .01 *P* = .890	*r* = −.09 *P* = .305	*r* = −.07 *P* = .447	*r* = −.09 *P* = .303
Transferrin *HFE*‐pos	*r* = .20 *P* = .118	*r* = −.30 *P* = .015	*r* = −.13 *P* = .315	*r* = −.13 *P* = .312	*r* = −.08 *P* = .556

Note

All partial correlations were controlled for age, sex, and education. Analyses involving QSM (quantitative susceptibility mapping) were additionally controlled for volume of putamen.

### 3.6 Associations between putaminal iron and performance as a function of *HFE* genotype

In the *HFE*‐neg group, the correlations between QSM in putamen and performance yielded only one significant association with working memory, with higher QSM being associated with lower performance (*r* = −.21, *P* = .013; bootstrapping 95% CI [−0.360 to −0.060]).

In the *HFE*‐pos group, a positive correlation was found between putaminal iron and executive function (*r* = 0.31, *P* = .016; bootstrapping 95% CI [0.085 to 0.508]), the other correlations being non‐significant (*Ps* > .19; Table 3, Figure 2).

When comparing *HFE* groups, the difference between correlations between putaminal QSM and working memory was at trend (*z* = 1.47, *P* = .07), whereas the correlations between QSM putamen and executive functioning were significantly different between groups (*z* = 2.44, *P* = .007).

### 3.7 Associations between Transf and performance as a function of *HFE* genotype

To limit the number of statistical tests, Transf was retained as the blood marker of iron because (1) *HFE* genotype had a significant effect on levels of Transf, and (2) it was significantly associated with putaminal iron. In the *HFE*‐neg group, Transf was not related to performance (*P*s > .21). In the *HFE*‐pos group, adjusting for the same covariates, a negative correlation was found between Transf and episodic memory (*r* = −.30, *P* = .015; bootstrapping 95% CI [−0.491 to −0.098]), but not with the other domains (*P*s > 0.11; Table 3, Figure 2).

The difference for the correlations between Transf and episodic memory between the two *HFE* groups was significant (*z* = 2.10, *P* = .018). Applying Bonferroni correction on the 20 performed partial correlations (corrected *P* = .05/20 = .0025), the three correlations with *P*‐values ranging from .013 to .016 within the *HFE* groups could only be considered trends, although the bootstrapping analyses supported the reliability of these associations.

### 3.8 Control analyses

Excluding the three participants who were either H63D homozygotes (N = 2) or heterozygous for both C282Y and H63D (N = 1) for hereditary hemochromatosis did not change any of the results. Moreover, these individuals were not outliers on any of the variables of interest.

Because of the presence of a few outliers (see Section 2.6, 3.8, Statistical Analyses), all concerned analyses were rerun after removing them. None of the results changed from what has been reported above, except for Iron: after removing the two identified outliers on the blood Iron data, the main effect of *HFE* status on Iron of the ANCOVA became significant (*P* = .019), such that the *HFE*‐pos group displayed higher Iron values than the *HFE*‐neg group. Regarding the significant correlation between working‐memory performance and QSM putamen in the *HFE*‐neg group, the 3 outliers identified within the working‐memory variable belonged to the *HFE*‐neg group; removing them led to a weakening of the correlation, but the trend remained (*r* = −.18, *P* = .035).

## 4 DISCUSSION

*HFE* H63D and/or C282Y mutations yielded higher levels of iron in putamen. Moreover, these polymorphisms significantly modulated blood markers of iron metabolism. However, older age did not magnify the combined genetic effect of C282Y and H63D on brain iron and blood markers of iron metabolism. Finally, relationships among putaminal iron, Transf, and cognitive performance differed as a function of *HFE* status.

Although participants were highly selected volunteers based on strict criteria, the frequencies of C282Y and H63D were comparable with previous reports. In our study, the percentage of 7.7% for C282Y is within the reported frequencies for North European populations, with reported figures between 5% and 10%.^48^, ^49^ In our sample, 24.5% of individuals were at least (and for the large majority) heterozygous for H63D, which is also in line with previously reported frequencies.^48^

### 4.1 *HFE* status effects on brain iron and blood markers of iron

In line with the literature, several blood markers of iron metabolism were altered according to *HFE* status. C282Y and/or H63D carriers displayed a typical pattern suggesting higher levels of iron such as increased Transf‐sat, reduced Transf, and Transf‐rec.^14^, ^15^, ^16^, ^17^, ^18^ Iron in putamen was increased in those carrying any or both mutations. This result supports previous studies where C282Y or H63D mutations were related to higher iron in the brain. For both C282Y and H63D *HFE* variants, Hagemeier et al^23^ found a moderate increase in putaminal iron in a sample of 150 individuals, an effect considered non‐significant after correction for false discovery rate. In the large UK Biobank neuroimaging sample, C282Y was most strongly related to higher iron in putamen, and H63D was related to higher striatal susceptibility.^20^, ^22^ By contrast, Bartzokis et al^21^ reported higher iron in caudate in a sample of 20 male carriers of either H63D and/or TfC2 compared with non‐carriers; finally, another study did not find any significant association of either *HFE* variants with brain iron in a sample of 314 individuals.^24^ All MRI methods for brain iron quantification are limited in terms of what is exactly measured at the biological level. This is also true for QSM, whose signal relies on tissue susceptibility. Nevertheless, it is assumed that QSM largely reflects brain iron concentration, as suggested by a post‐mortem validation study.^50^ The correlations we found between putaminal QSM and blood‐based markers of iron (Transf and Ferr) support the assumption that QSM indeed reflects iron concentration status.

### 4.2 Relation to age

Although increasing age was a significant factor of higher iron load in all investigated regions,^2^, ^6^ our results did not support the aging‐related magnification of genetic effects on the brain.^26^ Instead, our data suggest that these particular *HFE* mutations are invariantly associated with iron load across adulthood and do not trigger further iron overload in blood with increasing age, nor in the putamen, a region that typically shows one of the highest age‐related increase in iron load. Given the small sample size of the current study, more studies with bigger samples should be performed to confirm this finding.

### 4.3 Iron‐cognition relationships as a function of *HFE* status

A further aim of the present study was to investigate the relationships between brain iron, cognition and motor function, with the hypothesis that such relationships may differ according to *HFE* genotype. Based on growing evidence that higher iron load is generally deleterious to cognitive performance in older adults^6^, ^27^ and that C282Y/H63D is associated with disturbed iron metabolism leading to iron overload, the expectation that higher iron in the *HFE*‐pos group would be related to lower performance was reasonable. On the other hand, the limited literature on this topic has provided mixed findings, with some beneficial effects observed in *HFE*‐pos individuals. A handful of articles have suggested a possible advantage of being carrier of *HFE* mutations on several outcomes, such as better olfactory function, better physical endurance performance and aerobic capacity, and lower deleterious effect of pollution on cognition.^51^, ^52^, ^53^, ^54^ However, a meta‐analysis revealed a deleterious effect of being carrier of C282Y on memory performance.^55^ Our findings could only be considered trends after correction for multiple tests and therefore need to be interpreted cautiously. Our results concur with the counterintuitive hypothesis, namely a beneficial effect of being carrier of any or both C282Y or H63D for the association between iron and cognitive performance. More specifically, the *HFE*‐neg group displayed the expected pattern of higher iron related to poorer working‐memory performance, whereas the association was not significant in the *HFE*‐pos group. This result is remarkably similar to Bartzokis et al's findings, where the same pattern was found in non‐carriers vs. carriers of *HFE* H63D/*TfC2* in relation to working memory.^25^ In addition, our findings also supported a trend toward a positive association between putaminal iron and executive functioning in the *HFE*‐pos group, as well as a trend where lower Transf (which was negatively correlated with putaminal iron) was related to better episodic memory. These additional data tend to be in favor of an advantage of being carrier (most likely heterozygous) of C282Y or H63D.

Based on our and previous results, C282Y and H63D may exert beneficial effects in healthy individuals with low iron load, which may only become deleterious once a certain threshold of iron load is reached.^53^, ^56^ This theory has been discussed in relation to findings that placed the first occurrence and spread of the C282Y mutation centuries ago in some populations of Northern Europe, including Vikings, conferring advantages of increased body iron load in a challenging environment and living conditions.^11^, ^56^, ^57^ Our findings support the presence of a modulatory effect of *HFE* C282Y/H63D status on the relationships between brain iron load and cognitive performance and between blood markers of iron metabolism and cognition in healthy individuals. This seemingly beneficial effect of being carrier of C282Y or H63D requires more studies with larger samples to further evaluate the effects of *HFE* mutations on diverse outcomes in healthy individuals. It should be acknowledged that other mutations of *HFE* or other genes associated with HFE function and iron load (*HJV*, *HAMP*, and *TFR2*) likely contribute to brain iron load and may modulate the penetrance of *HFE* C282Y. However, we focused on common genetic variants of the *HFE* gene, well described in relation to disorders associated with iron overload.^58^, ^59^

## 5 CONCLUSION

Taken together, our findings suggest that *HFE* C282Y and H63D mutations contribute to increased brain iron content at the regional level, in addition to blood markers of iron metabolism, across adulthood. In terms of cognition, our results favor a possible advantage of higher blood and brain iron on cognition in healthy carriers of the C282Y and/or H63D *HFE* mutations. Independent replication studies in healthy populations are needed to confirm the observed associations.

## CONFLICT OF INTEREST

The authors of the present study declare that they have no conflict of interest.

## AUTHOR CONTRIBUTIONS

GK, FM, and GP conceived and designed the experiment. GK and FM collected the data. GK, FM, and GP performed the experiment. GK, FM, FF, EJL, and GP analyzed and interpreted the data. GK, FM, FF, EJL, and GP wrote the paper.

## APPROVAL OF THE RESEARCH PROTOCOL BY AN INSTITUTIONAL REVIEWER BOARD

The study was approved by the Regional Ethical Review Board in Stockholm (number 2016/457‐31/2) and conformed with provisions of the Declaration of Helsinki.

## INFORMED CONSENT

All participants signed informed consent prior to data collection.

## Data Availability

The data that support the findings of this study are available from the corresponding author upon reasonable request. The raw data belonged to the present study cannot be made publicly available, because the disclosure of personal data was not included in the research protocol and informed consent document.

## References

[1] Hare D, Ayton S, Bush A, Lei P. A delicate balance: iron metabolism and diseases of the brain. Front Aging Neurosci. 2013;5:34. 23874300 10.3389/fnagi.2013.00034 PMC3715022

[2] Daugherty A, Raz N. Age‐related differences in iron content of subcortical nuclei observed in vivo: a meta‐analysis. NeuroImage. 2013;70:113–21. 23277110 10.1016/j.neuroimage.2012.12.040 PMC3580001

[3] Daugherty AM, Raz N. Appraising the role of iron in brain aging and cognition: promises and limitations of MRI methods. Neuropsychol Rev. 2015;25(3):272–87. 26248580 10.1007/s11065-015-9292-y PMC4565753

[4] Daugherty AM, Raz N. Accumulation of iron in the putamen predicts its shrinkage in healthy older adults: a multi‐occasion longitudinal study. NeuroImage. 2016;128:11–20. 26746579 10.1016/j.neuroimage.2015.12.045 PMC4762718

[5] Ghadery C, Pirpamer L, Hofer E, Langkammer C, Petrovic K, Loitfelder M, et al. R2* mapping for brain iron: associations with cognition in normal aging. Neurobiol Aging. 2015;36(2):925–32. 25443291 10.1016/j.neurobiolaging.2014.09.013

[6] Kalpouzos G. Brain iron accumulation, and motor and cognitive decline in normal aging. Rev Neuropsychol. 2018;10(3):205–12.

[7] Kalpouzos G, Garzón B, Sitnikov R, Heiland C, Salami A, Persson J, et al. Higher striatal iron concentration is linked to frontostriatal underactivation and poorer memory in normal aging. Cereb Cortex. 2017;27(6):3427–36. 28334149 10.1093/cercor/bhx045

[8] Salami A, Avelar‐Pereira B, Garzón B, Sitnikov R, Kalpouzos G. Functional coherence of striatal resting‐state networks is modulated by striatal iron content. NeuroImage. 2018;183:495–503. 30125714 10.1016/j.neuroimage.2018.08.036

[9] Salami A, Papenberg G, Sitnikov R, Laukka EJ, Persson J, Kalpouzos G. Elevated neuroinflammation contributes to the deleterious impact of iron overload on brain function in aging. NeuroImage. 2021;230:e117792. 10.1016/j.neuroimage.2021.117792 33497770

[10] Zecca L, Youdim MB, Riederer P, Connor JR, Crichton RR. Iron, brain ageing and neurodegenerative disorders. Nat Rev Neurosci. 2004;5(11):863–73. 15496864 10.1038/nrn1537

[11] Barton JC, Edwards CQ, Acton RT. HFE gene: structure, function, mutations, and associated iron abnormalities. Gene. 2015;574(2):179–92. 26456104 10.1016/j.gene.2015.10.009 PMC6660136

[12] Corradini E, Buzzetti E, Pietrangelo A. Genetic iron overload disorders. Mol Aspects Med. 2020;75:e100896. 10.1016/j.mam.2020.100896 32912773

[13] Nandar W, Connor JR. HFE variants affect iron in the brain. J Nutri. 2011;141(4):729S–39S. 10.3945/jn.110.130351 21346098

[14] Bell S, Rigas AS, Magnusson MK, Ferkingstad E, Allara E, Bjornsdottir G, et al. A genome‐wide meta‐analysis yields 46 new loci associating with biomarkers of iron homeostasis. Commun Biol. 2021;4(1):156. 33536631 10.1038/s42003-020-01575-z PMC7859200

[15] Benyamin B, Esko T, Ried JS, Radhakrishnan A, Vermeulen SH, Traglia M, et al. Novel loci affecting iron homeostasis and their effects in individuals at risk for hemochromatosis. Nature Comm. 2014;5:4926. 10.1038/ncomms5926 PMC4215164 25352340

[16] Galesloot TE, Geurts‐Moespot AJ, den Heijer M, Sweep FC, Fleming RE, Kiemeney LA, et al. Associations of common variants in HFE and TMPRSS6 with iron parameters are independent of serum hepcidin in a general population: a replication study. J Med Genet. 2013;50(9):593–8. 23794717 10.1136/jmedgenet-2013-101673 PMC7784039

[17] Galesloot TE, Janss LL, Burgess S, Kiemeney LALM, den Heijer M, de Graaf J, et al. Iron and hepcidin as risk factors in atherosclerosis: what do the genes say? BMC Genet. 2015;16:79. 26159428 10.1186/s12863-015-0246-4 PMC4498499

[18] Jackson HA, Carter K, Darke C, Guttridge MG, Ravine D, Hutton RD, et al. HFE mutations, iron deficiency and overload in 10,500 blood donors. Br J Haematol. 2001;114(2):474–84. 11529872 10.1046/j.1365-2141.2001.02949.x

[19] Eckerström C, Frändberg S, Lyxe L, Pardi C, Konar J. Evaluation of a screening program for iron overload and *HFE* mutations in 50,493 blood donors. Ann Hematol. 2020;99(10):2295–301. 32844323 10.1007/s00277-020-04146-8 PMC7481153

[20] Atkins JL, Pilling LC, Heales CJ, Savage S, Kuo CL, Kuchel GA, et al. Hemochromatosis mutations, brain iron imaging, and dementia in the UK Biobank cohort. J Alzheimers Dis. 2021;79(3):1203–11. 33427739 10.3233/JAD-201080 PMC7990419

[21] Bartzokis G, Lu PH, Tishler TA, Peters DG, Kosenko A, Barrall KA, et al. Prevalent iron metabolism gene variants associated with increased brain ferritin iron in healthy older men. J Alzheimers Dis. 2010;20(1):333–41. 20164577 10.3233/JAD-2010-1368 PMC3119253

[22] Elliott LT, Sharp K, Alfaro‐Almagro F, Shi S, Miller KL, Douaud G, et al. Genome‐wide association studies of brain imaging phenotypes in UK Biobank. Nature. 2018;562(7726):210–6. 30305740 10.1038/s41586-018-0571-7 PMC6786974

[23] Hagemeier J, Ramanathan M, Schweser F, Dwyer MG, Lin F, Bergsland N, et al. Iron‐related gene variants and brain iron in multiple sclerosis and healthy individuals. Neuroimage Clin. 2017;17:530–40. 29201641 10.1016/j.nicl.2017.11.003 PMC5699896

[24] Pirpamer L, Hofer E, Gesierich B, De Guio F, Freudenberger P, Seiler S, et al. Determinants of iron accumulation in the normal aging brain. Neurobiol Aging. 2016;43:149–55. 27255824 10.1016/j.neurobiolaging.2016.04.002

[25] Bartzokis G, Lu PH, Tingus K, Peters DG, Amar CP, Tishler TA, et al. Gender and iron genes may modify associations between brain iron and memory in healthy aging. Neuropsychopharmacology. 2011;36(7):1375–84. 21389980 10.1038/npp.2011.22 PMC3096807

[26] Papenberg G, Lindenberger U, Bäckman L. Aging‐related magnification of genetic effects on cognitive and brain integrity. Trends Cogn Sci. 2015;19(9):506–14. 26187033 10.1016/j.tics.2015.06.008

[27] Spence H, McNeil CJ, Waiter GD. The impact of brain iron accumulation on cognition: a systematic review. PLoS One. 2020;15(10):e0240697. 33057378 10.1371/journal.pone.0240697 PMC7561208

[28] Gabriel S, Ziaugra L, Tabbaa D. SNP genotyping using the Sequenom MassARRAY iPLEX platform. Curr Protoc Hum Genet. 2009;60(1):1–18. 10.1002/0471142905.hg0212s60 19170031

[29] Ross P, Hall L, Smirnov I, Haff L. High level multiplex genotyping by MALDI‐TOF mass spectrometry. Nat Biotechnol. 1998;16(13):1347–51. 9853617 10.1038/4328

[30] Storm N, Darnhofer‐Patel B, van den Boom D, Rodi CP. MALDI‐TOF mass spectrometry‐based SNP genotyping. Methods Mol Biol. 2003;212:241–62. 12491915 10.1385/1-59259-327-5:241

[31] Liu T, Wisnieff C, Lou M, Chen W, Spincemaille P, Wang Y. Nonlinear formulation of the magnetic field to source relationship for robust quantitative susceptibility mapping. Magn Reson Med. 2013;69(2):467–76. 22488774 10.1002/mrm.24272

[32] Xu W, Cumming I. A region‐growing algorithm for InSAR phase unwrapping. IEEE Trans Geosci Remote Sens. 1999;37(1):124–34.

[33] Liu T, Khalidov I, de Rochefort L, Spincemaille P, Liu J, Tsiouris AJ, et al. A novel background field removal method for MRI using projection onto dipole fields (PDF). NMR Biomed. 2011;24(9):1129–36. 21387445 10.1002/nbm.1670 PMC3628923

[34] Liu J, Liu T, de Rochefort L, Ledoux J, Khalidov I, Chen W, et al. Morphology enabled dipole inversion for quantitative susceptibility mapping using structural consistency between the magnitude image and the susceptibility map. NeuroImage. 2012;59(3):2560–8. 21925276 10.1016/j.neuroimage.2011.08.082 PMC3254812

[35] Garzón B, Sitnikov R, Bäckman L, Kalpouzos G. Can transverse relaxation rates in deep gray matter be approximated from functional and T2‐weighted FLAIR scans for relative brain iron quantification? Magn Reson Imaging. 2017;40:75–82. 28438711 10.1016/j.mri.2017.04.005

[36] Andersson JLR, Jenkinson M, Smith S. Non‐linear Registration aka Spatial Normalisation – FMRIB Technical Report TR07JA2; 2007.

[37] Zhang Y, Brady M, Smith S. Segmentation of brain MR images through a hidden Markov random field model and the expectation‐maximization algorithm. IEEE Trans Med Imaging. 2001;20(1):45–57. 11293691 10.1109/42.906424

[38] Fischl B, Salat DH, Busa E, Albert M, Dieterich M, Haselgrove C, et al. Whole brain segmentation: automated labeling of neuroanatomical structures in the human brain. Neuron. 2002;33(3):341–55. 11832223 10.1016/s0896-6273(02)00569-x

[39] Fischl B, Salat DH, van der Kouwe AJW, Makris N, Ségonne F, Quinn BT, et al. Sequence‐independent segmentation of magnetic resonance images. NeuroImage. 2004;23(Suppl 1):S69–84. 15501102 10.1016/j.neuroimage.2004.07.016

[40] Fischl B, van der Kouwe A, Destrieux C, Halgren E, Ségonne F, Salat DH, et al. Automatically parcellating the human cerebral cortex. Cereb Cortex. 2004;14(1):11–22. 14654453 10.1093/cercor/bhg087

[41] Lecouvey G, Quinette P, Kalpouzos G, Guillery‐Girard B, Bejanin A, Gonneaud J, et al. Binding in working memory and frontal lobe in normal aging: is there any similarity with autism? Front Hum Neurosci. 2015;9:90. 25852510 10.3389/fnhum.2015.00090 PMC4362406

[42] Laukka EJ, Lövdén M, Herlitz A, Karlsson S, Ferencz B, Pantzar A, et al. Genetic effects on old‐age cognitive functioning: a population‐based study. Psychol Aging. 2013;28(1):262–74. 23276211 10.1037/a0030829

[43] Persson J, Kalpouzos G, Nilsson LG, Ryberg M, Nyberg L. Preserved hippocampus activation in normal aging as revealed by fMRI. Hippocampus. 2011;21(7):753–66. 20865729 10.1002/hipo.20794

[44] Lezak MD, Howieson DB, Loring DW, Hannay HJ, Fischer JS, editors. Neuropsychological Assessment, 4th edn. New York, NY: Oxford University Press; 2004.

[45] Baddeley AD. The capacity for generating information by randomization. Q J Exp Psychol. 1966;18(2):119–29. 5935121 10.1080/14640746608400019

[46] Bettcher BM, Libon DJ, Kaplan E, Swenson R, Penney DL. Digit symbol substitution test. In: Kreutzer SJ, DeLuca J, Caplan B, editors. Encyclopedia of Clinical Neuropsychology. New York, NY: Springer; 2011. p. 849–53.

[47] Tiffin J, Asher EJ. The Purdue pegboard: norms and studies of reliability and validity. J Appl Psychol. 1948;32(3):234–47. 18867059 10.1037/h0061266

[48] Hanson EH, Imperatore G, Burke W *HFE* gene and hereditary hemochromatosis: a HuGE review. Am J Epidemiol. 2001;154(3):193–206. 11479183 10.1093/aje/154.3.193

[49] Merryweather‐Clarke AT, Pointon JJ, Jouanolle AM, Rochette J, Robson KJ. Geography of HFE C282Y and H63D mutations. Genet Test. 2000;4(2):183–98. 10953959 10.1089/10906570050114902

[50] Langkammer C, Schweser F, Krebs N, Deistung A, Goessler W, Scheurer E, et al. Quantitative susceptibility mapping (QSM) as a means to measure brain iron? A post mortem validation study. Neuroimage. 2012;62:1593–9. 22634862 10.1016/j.neuroimage.2012.05.049 PMC3413885

[51] Grashow R, Sparrow D, Hu H, Weisskopf MG. Cumulative lead exposure is associated with reduced olfactory recognition performance in elderly men: the Normative Aging Study. Neurotoxicology. 2015;49:158–64. 26121922 10.1016/j.neuro.2015.06.006 PMC4523435

[52] Semenova EA, Miyamoto‐Mikami E, Akimov EB, Al‐Khelaifi F, Murakami H, Zempo H, et al. The association of HFE gene H63D polymorphism with endurance athlete status and aerobic capacity: novel findings and a meta‐analysis. Eur J Appl Physiol. 2020;120(3):665–73. 31970519 10.1007/s00421-020-04306-8 PMC7042188

[53] Thakkar D, Sicova M, Guest N, Garcia‐Bailo B, El‐Sohemy A. HFE genotype and endurance performance in competitive male athletes. Med Sci Sports Exerc. 2021;53(7):1385–90. 33433155 10.1249/MSS.0000000000002595

[54] Power MC, Weisskopf MG, Alexeeff SE, Wright RO, Coull BA, Spiro A III, et al. Modification by hemochromatosis gene polymorphisms of the association between traffic‐related air pollution and cognition in older men: a cohort study. Environ Health. 2013;12:16. 23413885 10.1186/1476-069X-12-16 PMC3599892

[55] Alfred T, Ben‐Shlomo Y, Cooper R, Hardy R, Deary IJ, Elliott J, et al. Genetic variants influencing biomarkers of nutrition are not associated with cognitive capability in middle‐aged and older adults. J Nutr. 2013;143(5):606–12. 23468552 10.3945/jn.112.171520 PMC3738233

[56] Hollerer I, Bachmann A, Muckenthaler MU. Pathophysiological consequences and benefits of HFE mutations: 20 years of research. Haematologica. 2017;102(5):809–17. 28280078 10.3324/haematol.2016.160432 PMC5477599

[57] Olsson KS, Konar J, Dufva IH, Ricksten A, Raha‐Chowdhury R. Was the C282Y mutation an Irish Gaelic mutation that the Vikings helped disseminate? HLA haplotype observations of hemochromatosis from the west coast of Sweden. Eur J Haematol. 2011;86(1):75–82. 20946107 10.1111/j.1600-0609.2010.01536.x

[58] Van Dijk BAC, Kemna EHJM, Tjalsma H, Klaver SM, Wiegerinck ETG, Goossens JP, et al. Effect of the new HJV‐L165X mutation on penetrance of HFE. Blood. 2007;109(12):5525–6. 17554070 10.1182/blood-2006-11-058560

[59] Sandhu K, Flintoff K, Chatfield MD, Dixon JL, Ramm LE, Ramm GA, et al. Phenotypic analysis of hemochromatosis subtypes reveals variations in severity of iron overload and clinical disease. Blood. 2018;132(1):101–10. 29743178 10.1182/blood-2018-02-830562

